# High Expression of microRNA-223 Indicates a Good Prognosis in Triple-Negative Breast Cancer

**DOI:** 10.3389/fonc.2021.630432

**Published:** 2021-04-13

**Authors:** Li Chen, Xiuzhi Zhu, Boyue Han, Lei Ji, Ling Yao, Zhonghua Wang

**Affiliations:** ^1^ Key Laboratory of Breast Cancer in Shanghai, Fudan University Shanghai Cancer Center, Shanghai, China; ^2^ Department of Breast Surgery, Fudan University Shanghai Cancer Center, Shanghai, China; ^3^ Department of Oncology, Shanghai Medical College, Fudan University, Shanghai, China

**Keywords:** microRNA-223, breast cancer, triple-negative, *in situ* hybridization, prognostic factor

## Abstract

**Purpose:**

MicroRNAs can influence many biological processes and have shown promise as cancer biomarkers. Few studies have focused on the expression of microRNA-223 (miR-223) and its precise role in breast cancer (BC). We aimed to examine the expression level of miR-223 and its prognostic value in BC.

**Methods:**

Tissue microarray (TMA)-based miRNA detection *in situ* hybridization (ISH) with a locked nucleic acid (LNA) probe was used to detect miR-223 expression in 450 BC tissue samples. Overall survival (OS) and disease-free survival (DFS) were compared between two groups using the Kaplan-Meier method and Cox regression model.

**Results:**

OS and DFS were prolonged in the high miR-223 expression group compared to the low miR-223 expression group (p < 0.0001 and p = 0.017, respectively), especially in patients with the triple-negative breast cancer (TNBC) subtype (p = 0.046 and p < 0.001, respectively). Univariate and multivariate Cox regression analyses revealed that TNM stage (p = 0.008), the molecular subtype (p = 0.049), and miR-223 (p < 0.001) were independently associated with OS and DFS. External validation was performed with the METABRIC and The Cancer Genome Atlas (TCGA) databases *via* online webtools and was consistent with the data described above.

**Conclusions:**

This study provides evidence that high miR-223 expression at diagnosis is associated with improved DFS and OS for BC patients, especially those with the TNBC subtype. miR-223 is a valid and independent prognostic biomarker in BC.

## Introduction

Breast cancer (BC) has overtook lung cancer as leading cause of cancer worldwide (https://www.iarc.who.int/wp-content/uploads/2021/02/pr294_E.pdf). In 2020, BC made up 11.7% of all new cancer cases globally, followed by lung cancer (11.4%) and colorectal cancer (10.0%). Due to the molecular heterogeneity of BC, individual biomarkers for BC are necessary (1). Since the groundbreaking work of Sørlie T and colleagues (2, 3) at the beginning of the 21st century, BC has been believed to consist of at least four different clinically relevant molecular subtypes, Luminal A, Luminal B, HER2 enriched, and triple-negative, classified according to the expression of estrogen receptor (ER), progesterone receptor (PR), human epidermal growth factor receptor 2 (HER2), and Ki-67. Although the PAM50 test, MammaPrint test, Oncotype DX test, and others have been used for clinical treatment and prognosis prediction (4), there are still no recognized clinically relevant biomarkers or effective therapeutic targets for BC except for ER, PR, and HER2 (5). Meanwhile, numerous new biomarkers are still being researched to help optimize personalized treatment in the management of BC, especially triple-negative breast cancer (TNBC), which has a high risk of recurrence and no effective targeted therapy (6, 7).

MicroRNAs (miRNAs) are a series of single-stranded, non-coding RNAs usually 21–25 nt in length (8). MiRNA expression profiling is gaining popularity because miRNAs, as key regulators in posttranscriptional gene expression networks, can influence many biological processes and have shown promise as biomarkers for cancer (9–11). In addition, miRNAs can generally be well preserved in body fluids, formalin-fixed and paraffin-embedded (FFPE) tissues, and other types of specimens for easy preservation and evaluation (9). Functional studies have confirmed that miRNA dysregulation is causal in many cancers, with miRNAs acting as tumor suppressors or oncogenes (12). The role of miRNAs in BC has been widely studied. Several oncogenic miRNAs are correlated with tumor aggressiveness and a poor prognosis, such as miR-221 (13), miR-301a (14), miR-493 (15), miR-200c, and miR-141 (16). Other tumor suppressor miRNAs, such as miR-34 (17, 18), miR-361-5p (19), miR-548p (20), and miR-205 (21, 22), can reduce migration and invasion capabilities upon their stably high expression.

MicroRNA-223 (miR-223), located at q12 of chromosome X, has been observed primarily in the myeloid lineage, especially neutrophils (23–27). To date, no consensus between the level of miR-223 and the type of disease or disease progression has been reached (28). In cancers, the role of miR-223 is conflicting. For example, the elevated expression of miR-223 is associated with a poor prognosis and drug resistance in non-small cell lung cancer (29) and gastric cancer (30, 31). In contrast, miR-223 plays a tumor suppressor role in human prostate cancer (32), bladder cancer (33), cervical cancer (34), and hepatocellular carcinoma (35, 36).

Until now, few studies have focused on the expression of miR-223 and its precise role in BC. Previous studies suggested that miR-223 improved treatment response in BC, including increasing the sensitivity to CDK4/6 inhibition (37) and suggesting a good response to radiotherapy (38). But none of them specifically focused on TNBC research. Xu Sun et al. demonstrated that miR-223 increased the sensitivity of TNBCs to apoptosis by targeting HAX-1 (39), but their study lacked clinical cohorts and specimens to validation. Therefore, we aimed to examine the expression level of miR-223 in our center’s well-developed tissue microarray (TMA) and its specific function and potential prognostic value in BC, especially TNBC.

## Materials and Methods

### Study Cohort

A total of 150 female patients diagnosed with stage I-III TNBC from August 2003 to November 2007 at Fudan University Shanghai Cancer Center (FUSCC; Shanghai, China) were consecutively recruited. According to the ratio approximately equal to 1.5 (ratio = TNBCs/other subtypes), 300 patients with other BC subtypes (Luminal A, Luminal B, and HER2-enriched) in the same period were randomly selected to avoid selection bias. All participants were diagnosed with invasive ductal carcinoma BC as the only primary tumor and underwent surgery, and their miR-223 expression levels were available. Overall survival (OS) was calculated as the time from the initial pathological diagnosis to death from any cause. Disease-free survival (DFS) was defined as the period from the initial pathological diagnosis to recurrence or metastasis or BC-specific death. The last follow-up time was March 2018, and the median follow-up time was 96.02 months.

Written permission was obtained for the collection of data from the FUSCC database. This study was ethically approved by the Ethical Committee and Institutional Review Board of FUSCC, and all the methods were performed in accordance with the approved guidelines.

### Tissue Microarrays (TMAs)

FFPE samples were obtained from the above BC patients before cancer treatment. The TMAs were constructed from FFPE samples by the Department of Pathology at FUSCC. Details on the development of the TMAs have been described in our previous studies (15, 19, 40–43). Briefly, an instrument was built for creating holes in the recipient array blocks and for acquiring tissue cores from the donor blocks (43). Stereotactic microscope was used to best select the areas of interest, with an additional bright light source under each block. After the block construction was completed, 8-μm sections of the resulting tumor tissue microarray block were cut with a microtome. An adhesive-coated tape system was a useful method for sectioning the tumor array blocks. The microtome knife cuts underneath a piece of tape that is placed over the block surface. The thin tissue section adheres to the tape, which is then rolled on an adhesive-coated microscope slide to transfer the section to the slide. Nearly all of the malignant tumors retain their histological pattern through the entire 3-mm-deep block. To reduce errors and improve accuracy, the TMAs were designed in duplicate cores in different areas of the same tumor.

### *In Situ* Hybridization (ISH)

ISH was performed on the TMAs using digoxigenin (DIG)-labeled miRCURY LNA robes from Exiqon (Vedbeak, Denmark) and an Enhanced Sensitive ISH Detection Kit I from Boster (Wuhan, China). The miR-223 probe sequence was 5′-TGGGGTATTTGACAAACTGAC-3′. Detailed ISH procedures have been described previously (15, 41). Briefly, TMAs were rewarmed at 65°C for 4 h, deparaffinized in xylene, and sequentially hydrated in gradient ethanol solutions (three times in 100%, once in 95%, once in 85%, and once in 75%). Then, TMAs were washed with PBS three times and incubated with 3% hydrogen peroxide for 10 min at room temperature. Next, TMAs were washed with 0.1% DEPC-H2O for 5 min, and then incubated with pepsin diluted 10-fold by citrate at 37°C for 20 min. After the digestion procedure exposing the nucleic acid fraction of RNA, TMAs were washed with PBS three times for 5 min each and with 0.1% DEPC-H2O once for 5 min. After incubation with prehybridization solution for 3 h at 37°C, the TMAs were incubated with 200 μl miRNA probe (20 nmol/L) that had been preheated for 10 min at 80°C and quickly transferred to an ice/water mixture for 5 min, in a hybridization box at 60°C overnight. The next day, TMAs were subjected to a stringent washing procedure with 2× saline sodium citrate (SSC), 0.5× SSC, and 0.2× SSC. After a 30-min wash in blocking solution, TMAs were sequentially incubated with biotinylated digoxin (60 min), streptavidin–biotin complex (20 min), and peroxidase (20 min) with a 5-min wash in 0.5 mol/L PBS between each. The results were visualized after staining with 3, 3-diaminobenzidine and counterstained with Gill hematoxylin.

### Staining Evaluation

**Figure 1:**
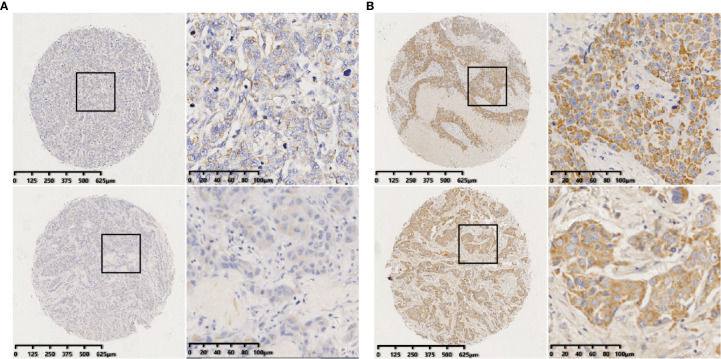
Identification of miR-223 in breast tumors by *in situ* hybridization (ISH). **(A)** Representative staining of negative miR-223 staining (staining index ≤4); **(B)** Representative staining of positive miR-223 staining (staining index >4) (scale bar of low-magnification = 125 μm and scale bar of high-magnification = 20 μm).

ISH staining was evaluated by two experienced pathologists independently in a blinded fashion. The staining index (SI) was used to incorporate the intensity and percentage of positive cells (15, 41). The intensity of staining was graded as follows: 0, no staining; 1, weak; 2, moderate; and 3, strong. The percentage of cells stained was graded as follows: 0, no staining; 1, <10%; 2, 10–50%; and 3, >50% tumor cells. The SI was calculated by multiplying the two scores. Samples with SIs >4 were defined as miR-223 high expression, whereas samples with SIs ≤4 were defined as miR-223 low expression. A third pathologist was consulted if there was a disagreement between the two observers. Examples of high and low expression stains are shown in **Figure 1**.

### miR-223 Expression in the METABRIC and TCGA Databases

The public data of miR-223 expression in the METABRIC and TCGA databases can be accessed online at Kaplan-Meier plotter https://kmplot.com/, which is a validation of survival biomarkers and capable of assessing the effect of 54k genes (mRNA, miRNA, and protein) on survival in 21 cancer types. A total of 1,262 BC patients from the METABRIC database and 1,078 BC patients from the TCGA database had complete follow-up data, pathological information, and miR-223 expression levels. All the details concerning how to use the online webtools have been described in the paper published by Lanczky et al. (44).

### Statistical Analysis

The relationships between miR-223 expression and clinicopathological parameters were based on Pearson’s χ2 tests or Fisher’s exact tests when necessary. OS and DFS were compared between the two groups using the Kaplan-Meier method and a Cox regression model. All p values were two-tailed, and a p value <0.05 was considered statistically significant. SPSS version 22.0 software (SPSS, Chicago, IL, USA) and R software version 3.5.3. (The R Project for Statistical Computing, https://www.r-project.org/) were used for the calculations and analyses. The R packages “survminer”, “readr”, and “survival” with the appropriate libraries were used. “Kaplan-Meier plotter” was utilized to test miR-223 as a biomarker of BC patient survival.

## Results

### Patient Characteristics and miR-223 Expression Patterns

For the 450 patients with invasive BC enrolled in this study, the median age was 51.31 years, and 49.1% of patients were menopausal. Among all patients, 55.11% were grade II and 26% were grade III. In addition, 56% (252/450) of patients had no lymph node metastasis, and 46.67% (210/450) had a tumor size ≤2 cm. Based on TNM stage, the proportions of patients in stage I, II, and III were 30.89, 54.67, and 11.11%, respectively. In the overall cohort, the four molecular subtypes (Luminal A, Luminal B, HER2-enriched, and triple-negative, definitions described in **Table 1**) accounted for 22, 24, 20.67, and 33.33%, respectively. In the staining evaluation, high staining for miR-223 was observed in 36.66% (165/450) of tumors. No significant associations were observed between miR-223 expression and clinicopathologic characteristics. Other related and detailed characteristics of the patients are shown in **Table 1**.

**Table 1:** Characteristics of the study cohort.

Variable	Number	miR-223 expression, number (%)		*P^a^* value
		Low	High	
**Total**	450	285	165	
**Age (years)**				0.399
≤40	52 (11.56%)	36 (8.00%)	16 (3.56%)	
40–60	330 (73.33%)	210 (46.67%)	120 (26.67%)	
>60	68 (15.11%)	39 (8.67%)	29 (6.44%)	
**Menopausal status**				0.438
pre	229 (50.89%)	149 (33.11%)	80 (17.78%)	
post	221 (49.11%)	136 (30.22%)	85 (18.89%)	
**Differentiation**				0.811
II	248 (55.11%)	155 (34.44%)	93 (20.67%)	
III	117 (26.00%)	77 (17.11%)	40 (8.89%)	
Missing	85 (18.89%)	53 (11.78%)	32 (7.11%)	
**Tumor size (cm)**				0.369
≤2	210 (46.67%)	131 (29.11%)	79 (17.56%)	
2–5	211 (46.89%)	133 (29.56%)	78 (17.33%)	
>5	20 (4.44%)	13 (2.89%)	7 (1.56%)	
Missing	9 (2.00%)	8 (1.78%)	1 (0.22%)	
**Lymph node status**				0.539
Negative	252 (56.00%)	153 (34.00%)	99 (22.00%)	
Positive	198 (44.00%)	132 (29.33%)	66 (14.67%)	
**ER**				0.622
Negative	255 (56.67%)	164 (36.44%)	91 (20.22%)	
Positive	195 (43.33%)	121 (26.89%)	74 (16.44%)	
**PR**				0.195
Negative	298 (66.22%)	195 (43.33%)	103 (22.89%)	
Positive	152 (33.78%)	90 (20.00%)	62 (13.78%)	
**HER2**				0.233
Negative	248 (55.11%)	151 (33.56%)	97 (21.56%)	
Positive	202 (44.89%)	134 (29.78%)	68 (15.11%)	
**TNM stage**				0.784
I	139 (30.89%)	84 (18.67%)	55 (12.22%)	
II	246 (54.67%)	157 (34.89%)	89 (19.78%)	
III	50 (11.11%)	34 (7.56%)	16 (3.56%)	
Missing	15 (3.33%)	10 (2.22%)	5 (1.11%)	
**Molecular subtype** ^b^				0.635
Luminal A	99 (22.00%)	58 (12.89%)	41 (9.11%)	
Luminal B	108 (24.00%)	72 (16.00%)	36 (8.00%)	
HER2-enriched	93 (20.67%)	61 (13.56%)	32 (7.11%)	
Triple-negative	150 (33.33%)	94 (20.89%)	56 (12.44%)	

ER, estrogen receptor; PR, progesterone receptor; HER2, human epidermal growth factor receptor 2.

a Based on Pearson’s χ2 test; Fisher’s exact test was used when needed.

b Definitions of subtypes: Luminal A (ER- and/or PR-positive, HER2-negative, PR high expression, and Ki-67 low expression), Luminal B (ER- and/or PR-positive, HER2-positive; ER-and/or PR-positive, HER2-negative, and Ki-67 high expression or PR low expression), HER2-enriched (ER- and PR-negative, HER2-positive), and triple-negative (ER-negative, PR-negative, and HER2-negative).

### Elevated miR-223 Expression Is Associated With Good Clinical Outcomes in BC Patients

**Figure 2:**
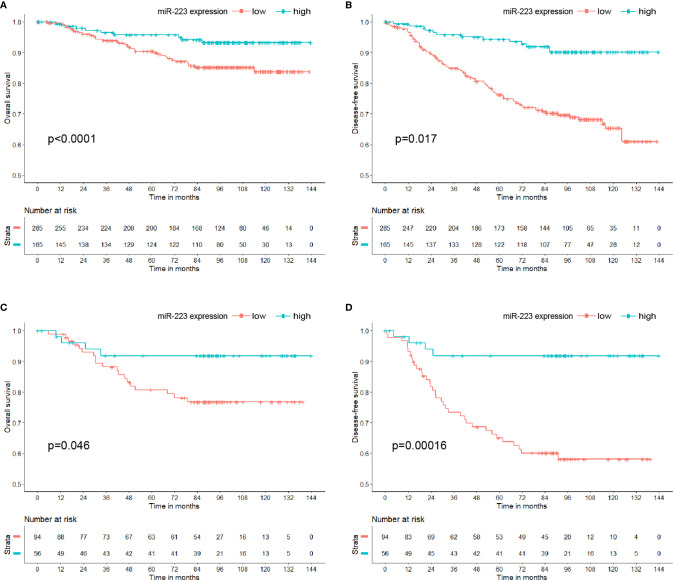
Kaplan-Meier curve of miR-223 expression in breast cancer patients. **(A)** Overall survival in breast cancer patients; **(B)** disease-free survival in breast cancer patients; **(C)** overall survival in triple-negative breast cancer patients; **(D)** disease-free survival in triple-negative breast cancer patients.

By the end of the study, a total of 20.22% (91/450) of patients experienced disease recurrence or metastasis, and 9.78% (44/450) died of BC. Among the 91 patients, 85.71% (78/91) had low miR-223 expression; among the 44 patients who died of BC, 79.55% (35/44) had low miR-223 expression. Kaplan-Meier analysis showed that the OS and DFS of the high miR-223 expression group were significantly better than those of the low miR-223 expression group (p < 0.001 and p = 0.017, respectively, **Figures 2A, B**). The 5-year DFS and OS rates of the low miR-223 expression group (76.24 and 90.43%, respectively) were significantly lower than those of the high miR-223 expression group (94.35 and 95.79%, respectively). Notably, both OS and DFS were significantly better in the high miR-223 TNBC group (p = 0.046 and p < 0.001, respectively, **Figures 2C, D**) and HER2-enriched group (p = 0.099 and p = 0.014, respectively, **Figures S1E, F**) than in the low miR-223 TNBC group, although the same trend was not observed in the other subtypes (**Figure S1**). In conclusion, high miR-223 expression indicates a better clinical outcome in BC patients, especially TNBC patients.

### Univariate and Multivariate Analyses of Prognostic Factors in BC Patients

Univariate and multivariate Cox regression analyses revealed that TNM stage (p = 0.008), the molecular subtype (p = 0.049), and miR-223 (p < 0.001) were independently associated with DFS (**Table 2**). The same factors were also determined to affect OS independently (**Table S1**). Since TNM stage overlapped with tumor size and lymph node status and the molecular subtype overlapped with ER, PR, and HER2 status, we did not incorporate TNM stage and the molecular subtype into the multivariate analyses to avoid study bias. The hazard ratios (HRs) of the low miR-223 expression group were 2.373 (95% CI: 1.140–4.937) for OS and 3.960 (95% CI: 2.201–7.126) for DFS, with the high miR-223 expression group used as a reference. All the results from the univariate analysis and multivariate Cox regression model are presented in **Tables 2** and **S1**.

**Table 2:** Univariate and multivariate analyses for disease-free survival.

	Univariate			Multivariate		
**Variable**	HR	95% CI	*P* value	HR	95% CI	*P* value
**Age**			0.594			
≤40 *vs >*60	1.351	0.626–2.914	0.444			
40–60 *vs >*60	0.994	0.548–1.803	0.984			
**Menopausal status**						
pre *vs* post	0.737	0.487–1.113	0.147			
**Differentiation**						
II *vs* III	0.730	0.457–1.164	0.186			
**TNM stage**			0.008			**0.008**
II *vs* I	1.421	0.860–2.346	0.170	1.485	0.887–2.485	0.132
III *vs* I	2.758	1.446–5.259	0.002	2.858	1.469–5.563	0.002
**Molecular subtype**			0.194			**0.049**
Luminal B *vs* Luminal A	1.106	0.580–2.107	0.760	0.813	0.415–1.593	0.546
HER2-enriched *vs* Luminal A	0.986	0.497–1.956	0.967	0.701	0.337–1.458	0.342
Triple-negative *vs* Luminal A	1.626	0.930–2.843	0.088	1.493	0.849–2.626	0.164
**miR-223**						
low *vs* high	3.960	2.201–7.126	<0.001	3.789	2.098–6.843	**<0.001**

CI, confidence interval; HR, hazard ratio.

The covariates in the Cox model were all categorical variables, and the adjusted p value and HR were derived from the model. The p value of the univariate analysis is marked bold.

In addition to the Cox prognosis, we explored the competing-risk nomogram. All of the validated factors in **Table 2** were incorporated to develop the competing-risks nomogram for predicting the 3-, 5-, and 10-year probability of DFS and OS by calculating the sum of the point values corresponding to each patient’s characteristics. **Figure S2** showed that expression of miR-223 was the strongest contributor to DFS, while TNM stage was the strongest contributor to OS. But unfortunately, we did not have external calibrations to verify it.

### Subgroup Analysis: miR-223 Is an Effective Predictive Factor of Clinical Outcomes in TNBC

In the subgroup analysis, miR-223 expression exhibited predictive potential for DFS and OS only in the TNBC subgroup (HR = 5.997, 95% CI 2.128–16.903, p = 0.001 for DFS; HR = 3.142, 95% CI 1.063–9.283, p = 0.038 for OS; **Table 3**) but not in the Luminal A (p = 0.217 for DFS, p = 0.277 for OS), Luminal B (p = 0.127 for DFS, p = 0.528 for OS), or HER2-enriched (p = 0.081 for DFS, p = 0.958 for OS) subgroup. Interestingly, among the 93 patients with HER2-enriched BC, five patients, all in the low miR-223 expression group, died, and no patient in the high expression group died. In addition, of the 15 patients who experienced relapse, 14 were in the low expression group, and only one was in the high expression group. This finding may explain why the statistics were slightly skewed. In summary, miR-223 is an effective predictor of clinical outcomes in TNBC, but it is worthy of further study in HER2-enriched BC.

**Table 3:** Multivariate Cox regression analysis for miR-223 as a prognostic marker for DFS and OS.

	DFS				OS		
Variable	HR	95% CI	*P* value		HR	95% CI	*P* value
**Molecular subtype**							
Luminal A	1.924	0.681–5.433	0.217		2.452	0.487–12.341	0.277
Luminal B	2.681	0.756–9.512	0.127		0.597	0.120–2.960	0.528
HER2-enriched	6.421	0.793–51.969	0.081		100402	0.000–6.4E+191	0.958
Triple-negative	5.997	2.128–16.903	**0.001**		3.142	1.063–9.283	**0.038**

CI, confidence interval; HR, hazard ratio; DFS, disease-free survival; OS, overall survival. The covariates in the Cox model included miR-223 and TNM stage. The adjusted p value and HR were derived from the Cox model; miR-223 = 1 was used as a reference. The p value of multivariate cox regression analysis for miR-223 in TNBC subtype is marked bold.

### External Validation in the METABRIC and TCGA Databases *via* Online Webtools

**Figure 3:**
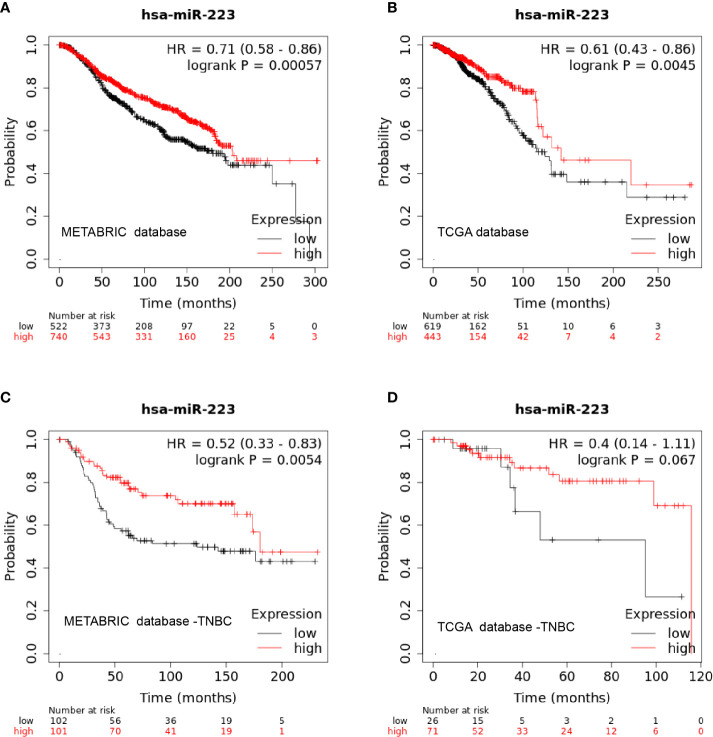
Kaplan-Meier curve of miR-223 expression in the METABRIC and TCGA databases. **(A)** overall survival in the METABRIC database; **(B)** overall survival in the TCGA database; **(C)** overall survival of triple-negative breast cancer patients in the METABRIC database; **(D)** overall survival of triple-negative breast cancer patients in the TCGA database.

To test the correlation between miR-223 expression and clinical prognosis, we performed survival analysis on data from publicly available datasets. As a survival biomarker discovery and validation tool based on a meta-analysis, Kaplan-Meier plotter is capable of assessing the effect of a miRNA on survival in BC patients. Data from Kaplan-Meier plotter showed that patients with high expression levels of miR-223 experienced a significantly longer OS time than those with low expression levels of miR-223 in both the METABRIC and TCGA databases (p < 0.001 and p = 0.0045, **Figures 3A, B**, respectively). In the detailed subtype analysis, we found that high miR-223 expression levels were associated with prolonged OS in patients with TNBC but not in those with the other subtypes based on data from the METABRIC database (p = 0.0054; **Figure 3C**), consistent with the results described above. The same trend was observed in the TCGA database, but there was no statistical significance (p = 0.067), which may be related to the deficiency of TNBC data (n = 98, **Figure 3D**).

The same results were also confirmed in patients with rectal adenocarcinoma (n = 160, p = 0.025), stomach adenocarcinoma (n = 436, p = 0.031), thymoma (n = 124, p = 0.03), and kidney renal papillary cell carcinoma (n = 291, p = 0.049); high expression levels of miR-223 were associated with prolonged OS (data not shown, available online at https://kmplot.com/).

## Discussion

Insights into the roles of miRNAs in disease development, particularly cancer, have made miRNAs attractive tools for novel biomarkers as well as therapeutic approaches (12). There is considerable evidence to indicate that miRNAs and their biogenesis machinery are involved in cancer development. The dysregulation of miRNA in tumors can be roughly divided into three parts. First, the dysregulation of miRNA biogenesis enzymes, such as downregulation of the miRNA biogenesis proteins Drosha and Dicer, is associated with poor patient outcomes (45–48). The other two points are the dysregulation of miRNAs with oncogenic function and the dysregulation of tumor suppressor miRNAs (described above). A mimic of the tumor suppressor miR-34 reached a phase I clinical trial (NCT01829971) for treating cancer. Currently, there is no universally recognized miRNA as a clinical prognostic biomarker or even as a clinical therapeutic target in BC. The search for effective biomarkers to guide treatment and predict prognosis is particularly important in TNBC, which is associated with high recurrence and lacks targeted therapy (49).

Our study is the first to evaluate the relationship between the expression of miR-223 and clinical outcomes in BC specimens. We found that the OS and DFS of the high miR-223 expression group were significantly better than those of the low miR-223 expression group, which was consistent with the results of some previous basic researches (37–39). The same results were also confirmed in the METABRIC and TCGA databases *via* online webtools. However, there was a study that differed from our results (50), which may be caused by different race, follow-up time, cohort size, as well as miR-223 detection methods. So far, no consensus has been reached between the expression level of miR-223 and the progression of the disease. This is also worthy of further study in the future. Surprisingly, no significant associations were observed between miR-223 expression and clinicopathologic characteristics. To a certain extent, miR-223 might be an important biomarker which was independent of these clinicopathological features, and needed to be paid attention to by clinical practice. The subsequent univariate and multivariate analyses confirmed that miR-223 is an independent prognostic factor for OS and DFS. Therefore, we speculate that miR-223 may be a protective factor in the prognosis of BC and can be detected to guide clinical practice in the future. Whether miR-223 is a tumor suppressor miRNA in BC requires further mechanistic research. In previous studies, miR-223 has been found to affect the cell cycle in acute myeloid leukemia by targeting the transcription factor E2F1 (51). Xu and coworkers found that miR-223 targets the tumor suppressor Fbxw7/Cdc4 (52). The latest research proved that miR-223 plays a key role in controlling steatosis-to-NASH progression by inhibiting two downstream targets, Cxcl10 and Taz (36).

In the subgroup analysis, the expression of miR-223 showed predictive potential for DFS and OS only in patients with TNBC but not in those with Luminal A, Luminal B, or HER2-enriched BC. According to the results from the METABRIC and TCGA database, miR-223 was also an effective predictive factor of clinical outcomes in TNBC. Regarding patients with HER2-enriched BC, DFS was better in the high miR-223 expression group, while statistical significance was not obtained for OS based on the small sample size. Unfortunately, miR-223 expression data on patients with HER2-enriched BC in the METABRIC and TCGA databases are less than our data. Interestingly, we analyzed miR-223 expression in the pan-cancer species *via* Kaplan-Meier plotter and found that high miR-223 expression was associated with a good prognosis in patients with rectal adenocarcinoma, stomach adenocarcinoma, thymoma, and kidney renal papillary cell carcinoma. Of course, this speculation needs further research and additional evidence.

In addition, several limitations to this study should be noted. First, our cohort is not representative of the composition of BC, and there were more patients with TNBC than with any of the other subtypes. Second, this was a retrospective analysis in a relatively small patient cohort. The use of the METABRIC and TCGA databases in the validation of the results might help attenuate these limitations. Third, we cannot explain the association between miR-223 and prognosis or why a high level of miR-223 indicates a good prognosis in adenocarcinoma but a poor prognosis in squamous cell carcinoma.

In conclusion, by using one of the largest BC TMAs available combined with a large public RNA sequencing database, this study provides evidence that high miR-223 expression at diagnosis is associated with prolonged DFS and OS in BC patients, especially those with the TNBC subtype. MiR-223 is a valid and independent prognostic biomarker in BC.

## Data Availability Statement

The original contributions presented in the study are included in the article/**Supplementary Material**. Further inquiries can be directed to the corresponding authors.

## Ethics Statement

All procedures performed in studies involving human participants were in accordance with the ethical standards of the institutional and/or national research committee and with the 1964 Helsinki declaration and its later amendments or comparable ethical standards. Informed consent was obtained from all individual participants included in the study.

## Author Contributions

ZW and LY designed the concept of the study. LC, LY, and XZ were responsible for data gathering. XZ and BH were responsible for statistical analysis and interpretation. LC and XZ wrote the final manuscript. LJ modified the manuscript. All authors contributed to the article and approved the submitted version.

## Funding 

This research was supported by the Natural Science Foundation of Shanghai (19ZR1411200) and Shanghai Anti-cancer Association (SACA-AX201904).

## Conflict of Interest

The authors declare that the research was conducted in the absence of any commercial or financial relationships that could be construed as a potential conflict of interest.

## Abbreviations

miR-223, microRNA-223; BC, breast cancer; TMA, tissue microarray; ISH, *in situ* hybridization; LNA, locked nucleic acid; OS, overall survival; DFS, disease-free survival; TNBC, triple-negative breast cancer; TCGA, The Cancer Genome Atlas; ER, estrogen receptor; PR, progesterone receptor; HER2, human epidermal growth factor receptor 2; FFPE, formalin-fixed and paraffin-embedded; FUSCC, Fudan University Shanghai Cancer Center; SSC, saline sodium citrate; SI, staining index; HRs, hazard ratios.
