# Supplementary material for: High Expression of microRNA-223 Indicates a Good Prognosis in Triple-Negative Breast Cancer
Source: Front Oncol. 2021 Apr 13;11:630432. doi: 10.3389/fonc.2021.630432 (PMC8078593; doi:10.3389/fonc.2021.630432)
Supplement: Supplementary file 1 [file DataSheet_1.doc]

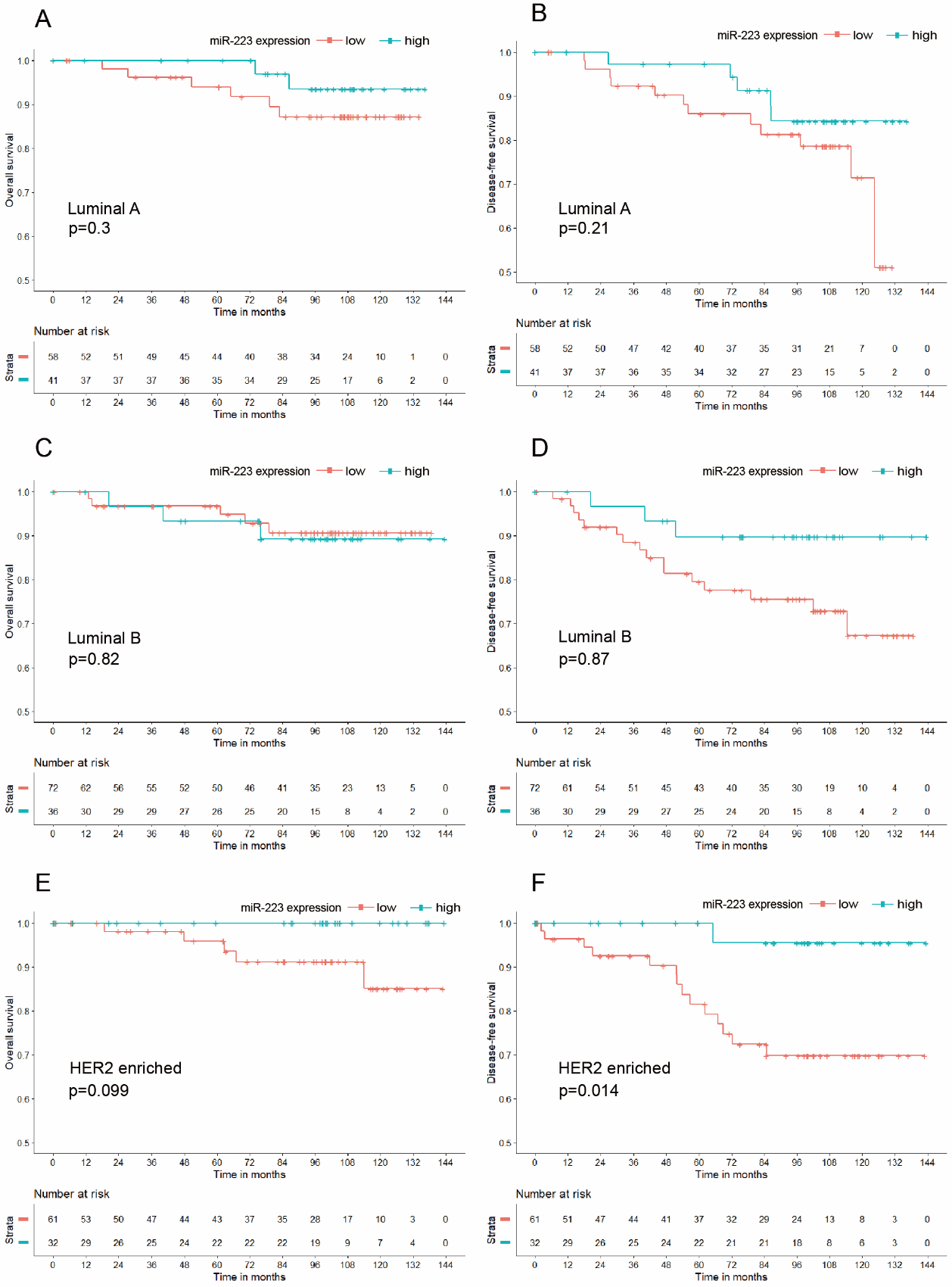


**Figure S1 Kaplan-Meier curve of miR-223 expression in breast cancer patients**

**(A)** Overall survival in Luminal A breast cancer patients; **(B)** disease-free survival in Luminal A breast cancer patients; **(C)** overall survival in Luminal B breast cancer patients; **(D)** disease-free survival in Luminal B breast cancer patients; **(E)** overall survival in HER2 enriched breast cancer patients; **(F)** disease-free survival in HER2 enriched breast cancer patients;


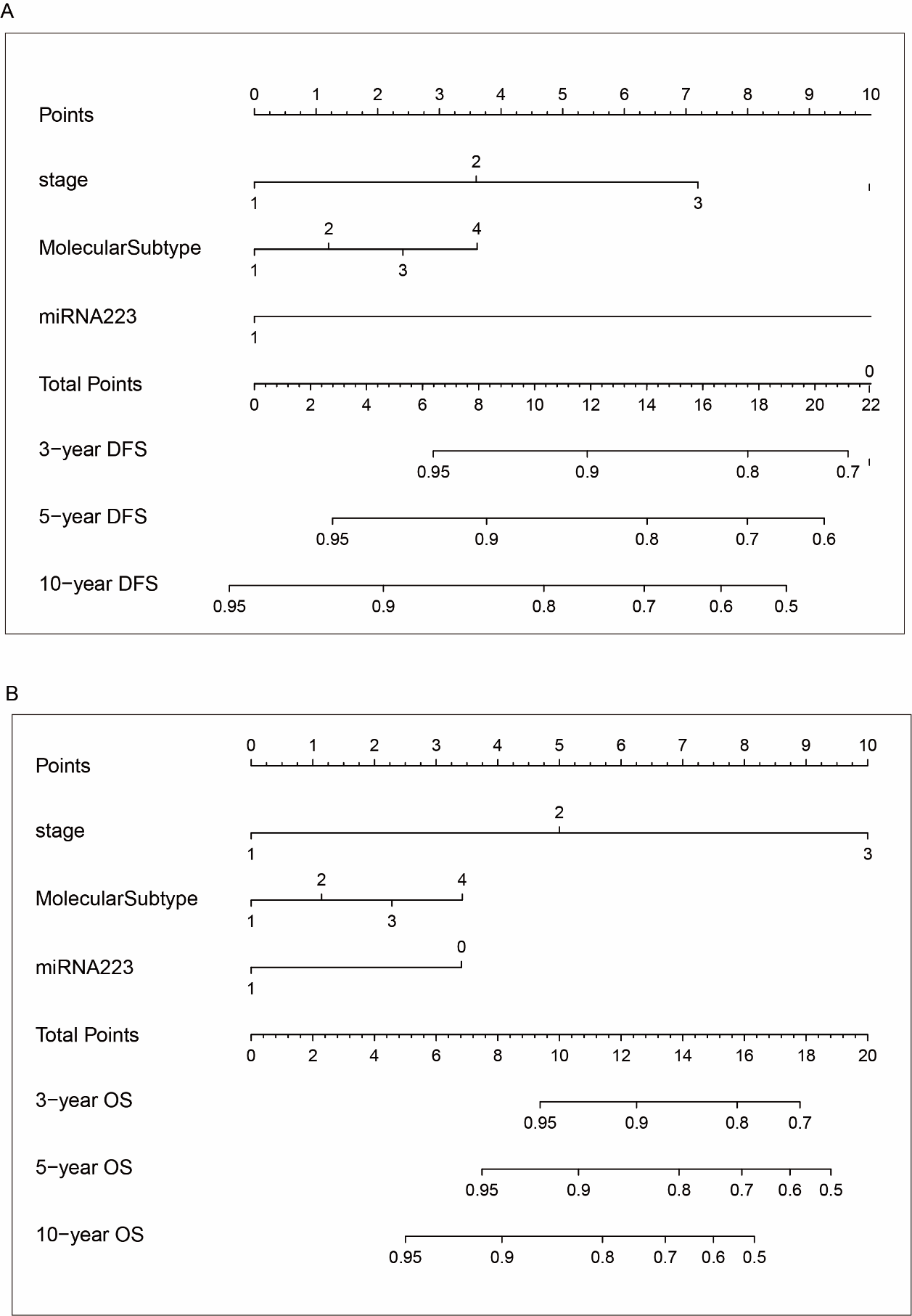


**Figure S2. Competing-risk nomogram.**

Competing-risk nomogram for predicting 3-, 5- and 10-year probabilities of **(A)** disease-free survival and **(B)** overall survival in breast cancer.

Abbreviations: DFS, disease-free survival; OS, overall survival.

| **Table S1 Univariate and multivariate analyses for overall survival** | | | | |  |  |
| --- | --- | --- | --- | --- | --- | --- |
|  | **Univariate** | | | **Multivariate** | | |
| **Variable** | HR | 95% CI | *P* value | HR | 95% CI | *P* value |
| **Age** |  |  | 0.268 |  |  |  |
| ≤40 vs >60 | 0.482 | 0.151-1.538 | 0.218 |  |  |  |
| 40-60 vs >60 | 0.577 | 0.282-1.181 | 0.132 |  |  |  |
| **Menopausal status** |  |  |  |  |  |  |
| pre vs post | 0.454 | 0.244-0.848 | 0.013 |  |  |  |
| **Differentiation** |  |  |  |  |  |  |
| II vs III | 0.456 | 0.239-0.870 | 0.017 |  |  |  |
| **TNM stage** |  |  | <0.001 |  |  | **<0.001** |
| II vs I | 2.449 | 1.001-5.993 | 0.050 | 2.728 | 1.103-6.743 | 0.030 |
| III vs I | 8.598 | 3.264-22.65 | <0.001 | 10.137 | 3.762-27.32 | <0.001 |
| IV vs I | 125.807 | 13.787-1147 | <0.001 | 283.078 | 24.648-3246 | <0.001 |
| **Molecular subtype** |  |  | 0.069 |  |  | **0.015** |
| Luminal B vs Luminal A | 1.030 | 0.387-2.745 | 0.953 | 0.626 | 0.228-1.717 | 0.362 |
| HER2-enriched vs Luminal A | 0.726 | 0.238-2.220 | 0.575 | 0.407 | 0121-1.369 | 0.146 |
| Triple-negative vs Luminal A | 2.044 | 0.914-4.571 | 0.082 | 1.677 | 0.745-3.774 | 0.212 |
| **miR-223** |  |  |  |  |  |  |
| low vs high | 2.373 | 1.140-4.937 | 0.021 | 2.309 | 1.100-4.849 | **0.027** |
| Abbreviations: CI, confidence interval; HR, hazard ratio. | | |  |  |  |  |
| The covariates in the Cox model were all categorical variables, and the adjusted *p* value and HR were derived from the model. | | | | | | |
